# Diffusion-Weighted Imaging Combined with Cervical Vascular Ultrasound in the Elderly Patients with Multiple Cerebral Infarction

**DOI:** 10.1155/2022/6461041

**Published:** 2022-03-31

**Authors:** Jinchun Lv, Jia Zhao

**Affiliations:** ^1^Radiology Department, Yueqing Hospital Affiliated to Wenzhou Medical University, Yueqing, Zhejiang 325600, China; ^2^Department of Ultrasound, Yueqing Hospital Affiliated to Wenzhou Medical University, Yueqing, Zhejiang 325600, China

## Abstract

**Objective:**

This study is aimed at evaluating the diagnostic value of diffusion-weighted imaging combined with cervical vascular ultrasound in the elderly patients with multiple cerebral infarctions and at demonstrating whether the diagnostic value is affected by the history of diabetes.

**Methods:**

From January 2020 to November 2021, the case data of 30 elderly patients with multiple cerebral infarction diagnosed in our hospital were included. Diffusion-weighted magnetic resonance imaging (DWI) and cervical vascular ultrasound (CAU) were performed, respectively. The diagnosis rates of the simple diffusion-weighted imaging group, the simple cervical vascular ultrasound group, and the diffusion-weighted imaging combined with cervical vascular ultrasound group were compared.

**Results:**

The median onset time of 30 patients was 26.5 (4.75, 43.0) hours. There were 10 hyperacute patients and 20 acute patients. The proportion of diagnosable patients in the diffusion-weighted imaging group was 83.8% (25/30), which was lower than the proportion of diagnosable patients in the diffusion-weighted imaging combined with cervical vascular ultrasound group, which was 90% (27/30). The difference was statistically significant (*χ*^2^ = 16.667; *P* < 0.001). The ratio of diagnosable patients in the cervical vascular ultrasound group alone was 66.7% (20/30), which was lower than 90% (27/30) in the diffusion-weighted imaging combined with cervical vascular ultrasound group. The difference was statistically significant (*χ*^2^ = 6.7; *P* = 0.010). In the hyperacute phase, the proportion of diagnosable patients in the diffusion-weighted imaging combined with cervical vascular ultrasound group was higher than that in the diffusion-weighted imaging group alone (*χ*^2^ = 5.833; *P* = 0.016) and the cervical vascular ultrasound group alone (*χ*^2^ = 2.500; *P* = 0.004). In the acute phase, the proportion of diagnosable patients in the diffusion-weighted imaging combined with cervical vascular ultrasound group was also higher those that in the diffusion-weighted imaging group alone (*χ*^2^ = 9.474; *P* = 0.002) and the cervical vascular ultrasound group alone (*χ*^2^ = 3.158; *P* = 0.006). The diagnostic accuracy of diffusion-weighted imaging combined with cervical vascular ultrasound was not significantly different between patients with history of diabetes and without history of diabetes (*χ*^2^ = 1.014; *P* = 0.314)

**Conclusion:**

The combined application of diffusion-weighted imaging and cervical vascular ultrasound has important value in improving the diagnosis rate of multiple cerebral infarction in the elderly, regardless of diabetes history, and it is worthy of clinical application.

## 1. Introduction

Cerebral infarction is the second most common cause of death and the third most common cause of disability worldwide, bringing heavy social and economic burdens [1]. Elderly patients are more prone to cerebral infarction, and multiple cerebral infarctions are more common [2]. The early diagnosis of elderly patients with multiple cerebral infarctions can be treated more quickly, which can help reduce the mortality and disability rates of patients with cerebral infarction and improve the prognosis [3]. Magnetic resonance is a technology based on the water content in tissues, and the diffusion-weighted imaging developed in recent years is a new type of magnetic resonance imaging method that can mainly measure and image the diffusion of water molecules, thus indirectly reflecting the characteristics of the microstructure of the organization [4]. The use of DWI has had a huge impact in both clinical and neuroimaging fields, and it has high sensitivity and specificity for the diagnosis of multiple cerebral infarction in the elderly [4]. However, DWI-negative multiple cerebral infarction limits the further promotion of this method [5]. Cervical vascular ultrasound (CAU) is a commonly used color doppler ultrasound examination in clinical practice. It can directly reflect the direction and shape of blood vessels and assess whether there is stenosis or occlusion in the lumen and has a high diagnostic value for cerebral infarction [6]. Diabetes, significantly associated with cerebral infarction, may expand the area of cerebral infarction and increase the risk of recurrence of cerebral infarction [7]. This study is aimed at evaluating the diagnostic value of diffusion-weighted imaging combined with CAU in the diagnosis of multiple cerebral infarctions in the elderly and at demonstrating whether the diagnostic value is affected by history of diabetes, providing a theoretical basis for the combined clinical application.

## 2. Materials and Methods

### 2.1. Participants

From January 2020 to November 2021, 30 elderly patients with multiple cerebral infarction were selected in our hospital, combined with the symptoms, signs, laboratory tests, and imaging examinations of the patients to confirm the diagnosis of multiple cerebral infarction. The average age of the 30 patients was 73.5 ± 3.8 years, including 16 males (53.3%) and 14 females (46.7%). Collect the patient's height, weight, time of onset, blood pressure, past medical history, and other data. Fasting venous blood was drawn to detect liver and kidney function, electrolytes, coagulation function, blood lipids, high-sensitivity C-reactive protein, and other indicators. Exclude patients who had a clear history of atrial fibrillation, valvular disease, severe liver and kidney disease, tumor, and other malignant diseases. All patients signed an informed consent form, and the research protocol was approved by the ethics review committee of Yueqing Hospital Affiliated to Wenzhou Medical University.

### 2.2. Imaging Methods

#### 2.2.1. Diffusion-Weighted Magnetic Resonance Imaging (DWI)

All patients were examined with the United Imaging uMR586 magnetic resonance instrument. An eight-channel special head coil was used for routine MR scanning and DWI scanning of the head. T1WI (T1-weighted imaging) and T2WI (T2-weighted imaging) were detected by the SE (spin echo) method and fast SE (fast spin echo) method, respectively. And DWI (diffusion-weighted imaging) was detected by the SS-EPI (single-shot echo planar imaging) method.

### 2.3. Cervical Vascular Ultrasound (CAU)

All patients underwent cervical vascular ultrasound examination, including bilateral subclavian, common cervical, external cervical, internal cervical, and vertebral arteries. The inner diameter of the patient's blood vessel and the thickness of the intima-media were measured to assess whether there was stenosis of the lumen, thickening of the intima, or carotid artery plaque.

### 2.4. Imaging Evaluation

Two senior radiologists and sonographers performed DWI and CAU assessments, respectively, to confirm the diagnosis of multiple cerebral infarction.

### 2.5. Statistical Analysis

Continuous variables were expressed as mean ± standard deviation or median (interquartile range). Categorical variables were expressed by frequency (rate), and the chi-square test was used for comparison between groups. All data were analyzed by IBM SPSS 21.0, and two-sided *P* < 0.05 was considered statistically significant.

## 3. Results

### 3.1. Clinical Characteristics of Elderly Patients with Multiple Cerebral Infarction

As shown in Table 1, the median onset time of the 30 participants was 26.5 (4.75, 43.0) hours and there were 10 hyperacute patients and 20 acute patients. The patients' average BMI, systolic blood pressure, and diastolic blood pressure were 24.5 ± 2.9 kg/m^2^, 136 ± 15 mmHg, and 71 ± 11 mmHg, respectively. The proportions of plaques found in previous diabetes, hypertension, and color doppler ultrasound were 23.3%, 46.7%, and 66.7%, respectively. The average alanine aminotransferase and aspartate aminotransferase of the participants were 37 ± 13 U/L and 33 ± 12 U/L, respectively, and the average blood creatinine and average low-density lipoprotein cholesterol were 56 ± 16 *μ*mol/L and 2.99 ± 0.65 mmol/L, respectively. The thickness of the carotid artery intima was 1.29 ± 0.29 cm.

### 3.2. Diffusion-Weighted Imaging Combined with Cervical Vascular Ultrasound in the Diagnosis of Multiple Cerebral Infarction in the Elderly

As shown in Table 2, the proportion of diagnosable patients in the diffusion-weighted imaging group alone was 83.8% (25/30), which was lower than the diagnosable proportion of patients in the diffusion-weighted imaging combined with cervical vascular ultrasound group, which was 90% (27/30). There was statistical significance (*χ*^2^ = 16.667; *P* < 0.001). The ratio of diagnosable patients in the cervical vascular ultrasound group alone was 66.7% (20/30), which was lower than 90% (27/30) in the diffusion-weighted imaging combined with cervical vascular ultrasound group. The difference was statistically significant (*χ*^2^ = 6.7; *P* = 0.010).

### 3.3. Diagnosis of Multiple Cerebral Infarction in the Elderly at Different Stages

As shown in Table 3, among patients in the hyperacute phase (<6 hours), the proportion of diagnosable patients in the DWI group alone was 70.0% (7/10), which was lower than the proportion of diagnosable patients in the combined group by 80% (8/10), and the difference was statistically significant (*χ*^2^ = 5.833; *P* = 0.016). The proportion of diagnosable patients in the CAU group alone was 50% (5/10), which was lower than the proportion of diagnosable patients in the combined group of 80% (8/10). The difference was statistically significant (*χ*^2^ = 2.500; *P* = 0.004). Among the patients in the acute phase (6 to 72 hours), the proportion of diagnosable patients in the DWI group alone was 90% (18/20), which was lower than the proportion of diagnosable patients in the combined group of 95% (19/20). The difference was statistically significant (*χ*^2^ = 9.474; *P* = 0.002). The proportion of diagnosable patients in the CAU group alone was 75% (15/20), which was lower than the proportion of diagnosable patients in the combined group of 95% (19/20). The difference was statistically significant (*χ*^2^ = 3.158; *P* = 0.006).

### 3.4. Diagnostic Value of Diffusion-Weighted Imaging Combined with Cervical Vascular Ultrasound Based on History of Diabetes

According to the history of diabetes, patients were divided into two groups: patients with history of diabetes and without diabetes history (Table 4). The diagnostic accuracy of diffusion-weighted imaging combined with cervical vascular ultrasound was 87% (20/23) in patients without diabetes and 100% (7/7) in patients with history of diabetes. The diagnostic performance of diffusion-weighted imaging combined with cervical vascular ultrasound was likely to be better in patients with history of diabetes than in patients without diabetes, but there was no significantly different among the two groups (*χ*^2^ = 1.014; *P* = 0.314).

## 4. Discussion

In this study, we found that in 30 elderly patients with multiple cerebral infarction, the combined use of magnetic resonance diffusion-weighted imaging and cervical vascular ultrasound can improve the diagnosis rate of cerebral infarction. Moreover, we also found that the diagnosis rate of the combined use of magnetic resonance diffusion-weighted imaging and cervical vascular ultrasound was better than that of diffusion-weighted imaging alone or cervical vascular ultrasound alone, regardless of whether it is in the hyperacute or acute phase.

Early diagnosis can reduce the number of dead brain cells and improve the severity of cerebral infarction. Timely diagnosis and treatment are essential to improve the prognosis of elderly patients with multiple cerebral infarction. Early diagnosis can reduce the mortality and disability rate of patients [8]. DWI is very sensitive to showing ischemic lesions of brain tissue, especially multiple acute ischemia, which is manifested as abnormal high signal in the lesion area, and the clarity and sensitivity of the display are better than conventional magnetic resonance [9]. Studies have shown that, compared with head CT, DWI has significantly higher sensitivity (88%-100%) and specificity (95%–100%) in the diagnosis of cerebral infarction [10]. However, it is worth noting that a meta-analysis study showed that the prevalence of DWI-negative cerebral infarction was 6.8% and DWI-negative cerebral infarction was significantly related to posterior circulation ischemia [5]. DWI-negative cerebral infarction not only is related to posterior circulation ischemia but may also be related to the following reasons: DWI may not be able to detect multiple small infarcts, especially multiple small infarcts that occur in the brain stem; in addition, DWI may not be able to detect some well hyperacute cerebral infarction [11].

Carotid vascular ultrasound, as a common clinically safe and effective examination, has the main value in diagnosing cerebral infarction in that it can accurately assess the characteristics, range, and location of intravascular plaques and show the degree of vascular stenosis and blockage [12]. In addition, according to the characteristics of the plaque shape, internal structure, and echo, it can be judged whether the plaque is stable. There are more lipid cells and inflammatory cells in unstable plaques, more irregular shapes on ultrasound, and more uneven echo [13]. The combined application of DWI and carotid vascular ultrasound can make up for each other's deficiencies, thereby increasing the diagnosis rate.

Diabetic patients have twice the risk of cerebral infarction than patients without diabetes, and diabetes is associated with the poor outcome after cerebral infarction [14]. Diabetes could accelerate the vascular aging of the brain, and it could increase the difficulty of accurate diagnosis of cerebral infarction [15]. However, in our study, the diagnostic accuracy of diffusion-weighted imaging combined with cervical vascular ultrasound is not significantly different between patients with history of diabetes and without diabetes.

The results of this study suggest that in the hyperacute and acute phases, the diagnosis rate of the combined group was superior to that of diffusion-weighted imaging alone or carotid vascular ultrasound alone. From the mechanism analysis, in the hyperacute phase of cerebral infarction, due to ischemic changes in the brain tissue, leading to cerebral edema, there is a high signal on DWI [16]. In the acute phase, the edema of the cerebral infarction area was aggravated and the extracellular space water increased and the DWI signal was significantly increased [16]. The combined use of DWI and cervical vascular ultrasound can effectively overcome the shortcomings of the two technologies in diagnosing hyperacute and acute multiple cerebral infarctions and better reflect the vascular lumen of the cerebral artery in patients with cerebral infarction and better evaluate the hemodynamics of blood vessels.

In short, the early diagnosis of multiple cerebral infarctions in the elderly is a clinically urgent problem. Based on the results of this study, we found that the combined use of magnetic resonance diffusion-weighted imaging and carotid vascular ultrasound can improve the diagnosis rate of multiple cerebral infarctions in the elderly.

## Figures and Tables

**Table 1:** Clinical characteristics of elderly patients with multiple cerebral infarction.

	People with cerebral infarction
Number of people, *n*	30
Age	73.5 ± 3.8
Proportion of males, *n* (%)	16 (53.3)
Onset time (h)	26.5 (4.75, 43.0)
Proportion of hyperacute phase, *n* (%)	10 (33.3)
Proportion in acute phase, *n* (%)	20 (66.7)
Body mass index (BMI) (kg/m^2^)	24.5 ± 2.9
Systolic blood pressure (mmHg)	136 ± 15
Diastolic blood pressure (mmHg)	71 ± 11
History of diabetes, *n* (%)	7 (23.3)
History of hypertension, *n* (%)	14 (46.7)
Alanine aminotransferase (U/L)	37 ± 13
Aspartate aminotransferase (U/L)	33 ± 12
Serum creatinine (*μ*mol/L)	56 ± 16
Low-density lipoprotein cholesterol (mmol/L)	2.99 ± 0.65
High sensitivity C-reactive protein (mg/L)	4.5 ± 2.6
Carotid artery intima thickness (cm)	1.29 ± 0.29
Proportion of carotid artery plaque, *n* (%)	20 (66.7)

**Table 2:** Diffusion-weighted imaging combined with cervical vascular ultrasound in the diagnosis of multiple cerebral infarction in the elderly.

Inspection method	Simple diffusion-weighted imaging	Simple cervical vascular ultrasound	Diffusion-weighted imaging combined with cervical vascular ultrasound
Total number of confirmed cases, *n*	30	30	30
Number of people diagnosed, *n*	25	20	27
Percentage (%)	83.8	66.7	90
*χ* ^2^	16.667^∗^	6.7^#^	
*P*	<0.001	0.010	

^∗^Comparison between the diffusion-weighted imaging group and the diffusion-weighted imaging combined with cervical vascular ultrasound group. ^#^Comparison between the cervical vascular ultrasound group and the diffusion-weighted imaging combined with cervical vascular ultrasound group.

**Table 3:** Diagnosis results of multiple cerebral infarction in the elderly at different stages.

Inspection method	Total number of people diagnosed, *n*	Number of people diagnosed, *n*	Percentage (%)	Chi-square value	*P* value
Hyperacute phase					
Simple diffusion -weighted imaging	10	7	70	5.833^∗^	0.016
Simple cervical vascular ultrasound	10	5	50	2.500^#^	0.004
Diffusion-weighted imaging combined with cervical vascular ultrasound	10	8	80	—	—
Acute phase					
Diffusion-weighted imaging	20	18	90	9.474^∗^	0.002
Cervical vascular ultrasound	20	15	75	3.158^#^	0.006
Diffusion-weighted imaging combined with cervical vascular ultrasound	20	19	95	—	—

^∗^Comparison between the diffusion-weighted imaging group and the diffusion-weighted imaging combined with cervical vascular ultrasound group. ^#^Comparison between the cervical vascular ultrasound group and the diffusion-weighted imaging combined with cervical vascular ultrasound group.

**Table 4:** Diagnostic value of diffusion-weighted imaging combined with cervical vascular ultrasound based on the history of diabetes^∗^.

		Diffusion-weighted imaging combined with cervical vascular ultrasound		Total
		Negative	Positive	
History of diabetes	No	3 (13)	20 (87)	23 (100)
	Yes	0 (0)	7 (100)	7 (100)
Total		3 (10)	27 (90)	30 (100)

The data was expressed as *n* (%). ^∗^The chi-square value was 1.014, and the *P* value was 0.314.

## Data Availability

The analyzed datasets generated during the study are available from the corresponding author upon reasonable request.

## References

[1] Feigin V. L., Norrving B., Mensah G. A. (2017). Global burden of stroke. *Circulation Research*.

[2] Zhou M., Wang H., Zeng X. (2019). Mortality, morbidity, and risk factors in China and its provinces, 1990-2017: a systematic analysis for the Global Burden of Disease Study 2017. *The Lancet*.

[3] Pandian J. D., Gall S. L., Kate M. P. (2018). Prevention of stroke: a global perspective. *The Lancet*.

[4] Guadilla I., Calle D., Lopez-Larrubia P. (2018). Diffusion-weighted magnetic resonance imaging. *Methods in Molecular Biology*.

[5] Edlow B. L., Hurwitz S., Edlow J. A. (2017). Diagnosis of DWI-negative acute ischemic stroke: a meta-analysis. *Neurology*.

[6] Naidich J. B., Weiss A., Grimaldi G. M., Kohn N., Naidich J. J., Pellerito J. S. (2018). Carotid ultrasound examinations. *Ultrasound Quarterly*.

[7] Chen R., Ovbiagele B., Feng W. (2016). Diabetes and stroke: epidemiology, pathophysiology, pharmaceuticals and outcomes. *The American Journal of the Medical Sciences*.

[8] Saver J. L. (2006). Time is brain—quantified. *Stroke*.

[9] Nagaraja N., Forder J. R., Warach S., Merino J. G. (2020). Reversible diffusion-weighted imaging lesions in acute ischemic stroke: a systematic review. *Neurology*.

[10] Jauch E. C., Saver J. L., Adams H. P. (2013). Guidelines for the early management of patients with acute ischemic stroke. *Stroke*.

[11] Kawano H., Hirano T., Nakajima M., Inatomi Y., Yonehara T. (2013). Diffusion-weighted magnetic resonance imaging may underestimate acute ischemic lesions: cautions on neglecting a computed tomography-diffusion-weighted imaging discrepancy. *Stroke*.

[12] Murray C. S. G., Nahar T., Kalashyan H., Becher H., Nanda N. C. (2018). Ultrasound assessment of carotid arteries: current concepts, methodologies, diagnostic criteria, and technological advancements. *Echocardiography*.

[13] Latha S., Samiappan D., Kumar R. (2020). Carotid artery ultrasound image analysis: a review of the literature. *Proceedings of the Institution of Mechanical Engineers Part H*.

[14] Ergul A., Li W., Elgebaly M. M., Bruno A., Fagan S. C. (2009). Hyperglycemia, diabetes and stroke: focus on the cerebrovasculature. *Vascular Pharmacology*.

[15] Hill M. D. (2014). Stroke and diabetes mellitus. *Handbook of Clinical Neurology*.

[16] Gonzalez R. G., Schaefer P. W., Buonanno F. S. (1999). Diffusion-weighted MR imaging: diagnostic accuracy in patients imaged within 6 hours of stroke symptom onset. *Radiology*.

